# Metronomic Treatment with Low-Dose Trofosfamide Leads to a Long-Term Remission in a Patient with Docetaxel-Refractory Advanced Metastatic Prostate Cancer

**DOI:** 10.1155/2010/395720

**Published:** 2010-04-11

**Authors:** Jochen Greiner, Rainer Küfer, Sven N. Reske, Volker Martin, Hartmut Döhner, Mark Ringhoffer

**Affiliations:** ^1^Department of Internal Medicine III, University of Ulm, Albert Einstein-Allee 23, 89081 Ulm, Germany; ^2^Department of Urology, University of Ulm, Robert-Koch-Str. 8, 89081 Ulm, Germany; ^3^Department of Nuclear Medicine, University of Ulm, Robert-Koch-Str. 8, 89081 Ulm, Germany; ^4^Internal Medicine, Clinic of Dinkelsbühl, Germany; ^5^IIIrd Department of Medicine, Clinic of Karlsruhe, 76133 Karlsruhe, Germany

## Abstract

The treatment of metastatic prostate cancer patients refractory to androgen withdrawal and docetaxel therapy is currently discouraging and new therapeutic approaches are vastly needed. Here, we report a long-term remission over one year in a 68-year-old patient with metastatic docetaxel-refractory prostate cancer employing low-dose trofosfamide. The patient suffered from distant failure with several bone lesions and lymph node metastases depicted by a (11) C-Choline positron emission tomography/computerized tomography (PET/CT). After initiation of trofosfamide 100 mg taken orally once a day we observed a steadily decreasing PSA value from initial 46.6 down to 2.1 *μ*g/L. The Choline-PET/CT was repeated after 10 months of continuous therapy and demonstrated a partial remission of the bone lesions and a regression of all involved lymph nodes but one. Taken together we found an astonishing and durable activity of the alkylating agent trofosfamide given in a metronomic fashion. We rate the side effects as low and state an excellent therapeutic ratio of this drug in our patient.

## 1. Introduction

In hormone-refractory prostate cancer (HRPC) the treatment of choice is chemotherapy with docetaxel plus prednisone since this combination has shown a survival benefit in a large phase III trial. However, this therapy is often associated with considerable toxicity. After docetaxel failure currently no standard of care exists. In such a palliative setting a treatment strategy is highly desirable, which provides antitumoral activity without causing a significant amount of toxicity. In the last years the concept of metronomic chemotherapy has emerged, which fulfills these requirements [1]. Metronomic chemotherapy means the continuous administration of relatively low, nontoxic doses of chemotherapeutic drugs mainly aiming on the prevention of tumor angiogenesis. Alkylating agents like cyclophosphamide and the more lipophilic trofosfamide have been employed in this therapeutic setting and have led to surprisingly potent tumor effects in a wide range of tumors [2, 3]. Here we report on patient with advanced prostate cancer, who achieved a long-term remission after therapy with low-dose trofosfamide. The present case demonstrates the value of the metronomic concept in patients with advanced HRPC.

## 2. Case Presentation

In this paper, we describe a long-term partial remission over one year of a 68-year-old man treated with oral trofosfamide. Diagnosis was established in January 2005 (pT3b pN1 M0 V1 L1, Gleason Score 9) and prostatectomy, extended field radiation, and orchiectomy were performed. After complete androgen depletion with bicalutamide (casodex) the patient relapsed one year later. This patient showed multiple bone lesions and lymph node metastases and was refractory to standard dose docetaxel treatment (75 mg/m^2^, qd21, six cycles). After the beginning of docetaxel application PSA was initially declining then increased again. Moreover, after six cycles of docetaxel the patient developed a polyneuropathy grade II. Directly after docetaxel treatment the PSA value rose continuously and antihormonal treatment was ongoing. Before and after treatment with docetaxel, alkaline phosphatase, haemoglobin, and lactate dehydrogenase were in normal ranges. Intravenous bisphosphonate treatment with zoledronic acid (4 mg, every four weeks) was already started one year before the initiation of trofosfamide therapy. 

**Figure 1:**
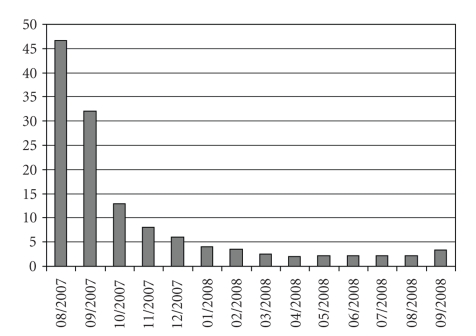
PSA course during trofosfamide treatment.

**Figure 2:**
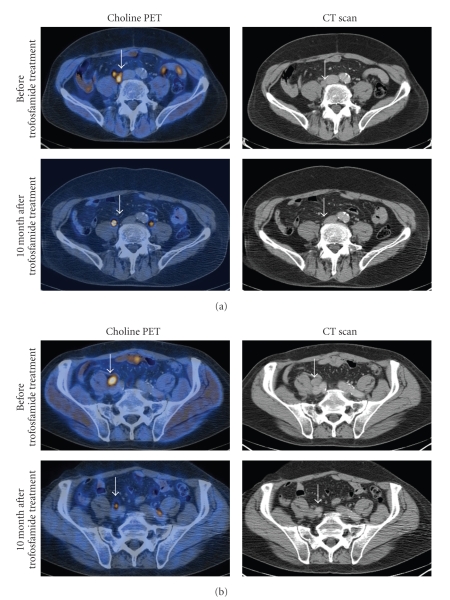
(a) and (b) show exemplarily the regression of a lymph node metastasis, demonstrated by choline-PET-CT before and 10 months after treatment with trofosfamide.

Trofosfamide was started in August 2007 with the standard dose of 150 mg orally per day in an outpatient setting. Due to moderate side effects like a facial rash and paresthesia of the lower legs, the dose was reduced to 100 mg per day after 4 weeks of treatment. Both side effects are not typical for trofosfamide. Probably the neurological symptoms can be explained with preexisting polyneuropathy which already developed during the docetaxel treatment. The neuropathy was under excellent control by a treatment with pregabalin. After treatment the PSA value continuously decreased from 46.6 to 2.1 *μ*g/L. Figure 1 illustrates the PSA course before and after a one-year treatment period with trofosfamide. No significant treatment-related toxicity was detected after the dose reduction to 100 mg and the drug was tolerated well (Karnofsky index 100% during the whole treatment). (11) C-choline positron emission tomography/computerized tomography (PET/CT) was performed before and 10 months after start of treatment with trofosfamide. In the choline-PET/CT a partial remission of bone lesions and a regression of all but one involved lymph nodes were demonstrated. Figures 2(a) and 2(b) show the remission of systemic metastases detected by choline-PET/CT after a 10-month treatment with trofosfamide.

## 3. Discussion

Prostate cancer is a common cause of death in men and remains incurable in the metastatic setting. Standard treatment for metastatic disease contains androgen ablation followed by chemotherapy, if hormone-refractoriness occurs. Chemotherapy has evolved over time since the 1960s and several agents are currently approved like estramustine, mitoxantrone, and docetaxel. Two important clinical trials (TAX 327 and SWOG 99-16) showed for the first time a survival benefit in men with metastatic HRPC. In particular, docetaxel-based chemotherapy demonstrated a median improvement in overall survival of 2.5 months as compared with mitoxantrone and prednisone (MP) in metastatic HRPC [4]. If a patient is refractory to docetaxel, he can proceed to investigational therapy. In this situation second-line chemotherapy has not been extensively studied, but generally it leads to a short median progression-free survival of just a few months. Rosenberg et al. demonstrated a modest activity of second-line chemotherapy (phase 2 study of ixabepilone or mitoxantrone and prednisone) with no advantage for any treatment arm [5]. 

A promising new treatment strategy in prostate cancer is the targeting of tumor angiogenesis. Antiangiogenic strategies seem to be rationale in HRPC, because microvessel density is an independent prognostic factor for progression and survival in clinically localized prostate cancer. Moreover, tumor angiogenesis correlates with metastases and progression also in advanced HRPC [6, 7]. Several new drugs, which have antiangiogenic activity like bevacizumab, tyrosine kinase inhibitors like sorafenib or sunitinib, and mTor inhibitors like everolimus are under clinical investigation. These targeted agents either neutralize VEGF directly or abrogate downstream pathways of growth receptors like VEGFR or PDGFR. 

Beside targeted drugs, thalidomide and low-dose trofosfamide are also effective through an antiangiogenic manner; however, they employ a different mode of action [8]. In a study including twenty patients with a progression on androgen ablation and/or estramustine treated with trofosfamide per os (150 mg/day) the authors concluded that trofosfamide has activity in hormone-refractory advanced prostate cancer, 5 patients (27%) had a decline of the PSA levels [9]. In contrast to a cyclic schedule, where the dosage intervals lead to peak levels of the cytostatic drugs followed by subtherapeutic levels, continuous application of the active drug maintains a more constant drug level. As a consequence, metronomic scheduling of trofosfamide prevented the mobilization of circulating endothelial progenitor cells (EPCs). This was in contrast to conventional dose-dense chemotherapy which promoted a release of EPCs through a rebound mechanism [10]. Although we have no transitional data to support the hypothesis in this case, one might speculate that such a prevention of EPCs release might have also contributed to the favorable clinical course of our patient, who received 50 mg per day less than the standard dose. It is noteworthy that the combined use of antiangiogenic agents and zoledronic acid seems to put the patients at risk of a characteristical bisphosphonate-associated complication named osteonecrosis of the jaw (ONJ) [11] and one should have this complication in mind when clinical signs occur.

## 4. Conclusion

Taken together, we found high activity of metronomically administered trofosfamide in a situation beyond standard treatment algorithms. We conclude, that low-dose trofosfamide could be a treatment option for patients with advanced prostate cancer, also in a docetaxel-resistant setting. Since our patient improved with a lowered dose of trofosfamide, we suggest that the optimal antiangiogenic dose and schedule of trofosfamide in this setting has yet to be determined.
